# Advances in the study of miRNAs in chronic kidney disease with cardiovascular complications

**DOI:** 10.3389/fphys.2023.1283597

**Published:** 2023-11-23

**Authors:** Chenchen Zou

**Affiliations:** Department of Geriatrics, Jiangyin Hospital of Traditional Chinese Medicine, Wuxi, Jiangsu, China

**Keywords:** chronic kidney disease, cardiovascular disease, miRNAs, advanced manufacturing, study

## Abstract

Chronic kidney disease (CKD) is characterised by gradual loss of renal function and cardiovascular disease (CVD) as its principal consequence. CVD is a substantial source of morbidity and death in the CKD population and a growing global concern. Because there are no reliable early biomarkers to follow the progression of CKD and predict the risk of complications, research into such molecules continues. Many studies have demonstrated that miRNAs are potentially important variables in CKD, are very stable in blood, and may be employed as diagnostic and prognostic markers for various disorders. Vascular calcification (VC) is a cell-mediated process that necessitates genetic defects in the combined cardiovascular issues of CKD and may be modulated in part by miRNAs. Numerous miRNAs have been linked to the progression of vascular calcification. Many miRNAs have been discovered as being important in ventricular hypertrophy, including miRNA-30, miRNA-212, and miRNA-133. Endothelium miR-126, miR-92a-3p, and others are important regulators of angiogenesis, endothelium repair, and homeostasis. Several interesting non-invasive miRNA biomarkers in CKD/CVD have been found, with the potential to enhance diagnostic accuracy, predict prognosis, track disease progression, and serve as novel therapy targets. However, large-scale clinical studies are still needed to determine the therapeutic utility of miRNA.

## Introduction

**FIGURE 1:**
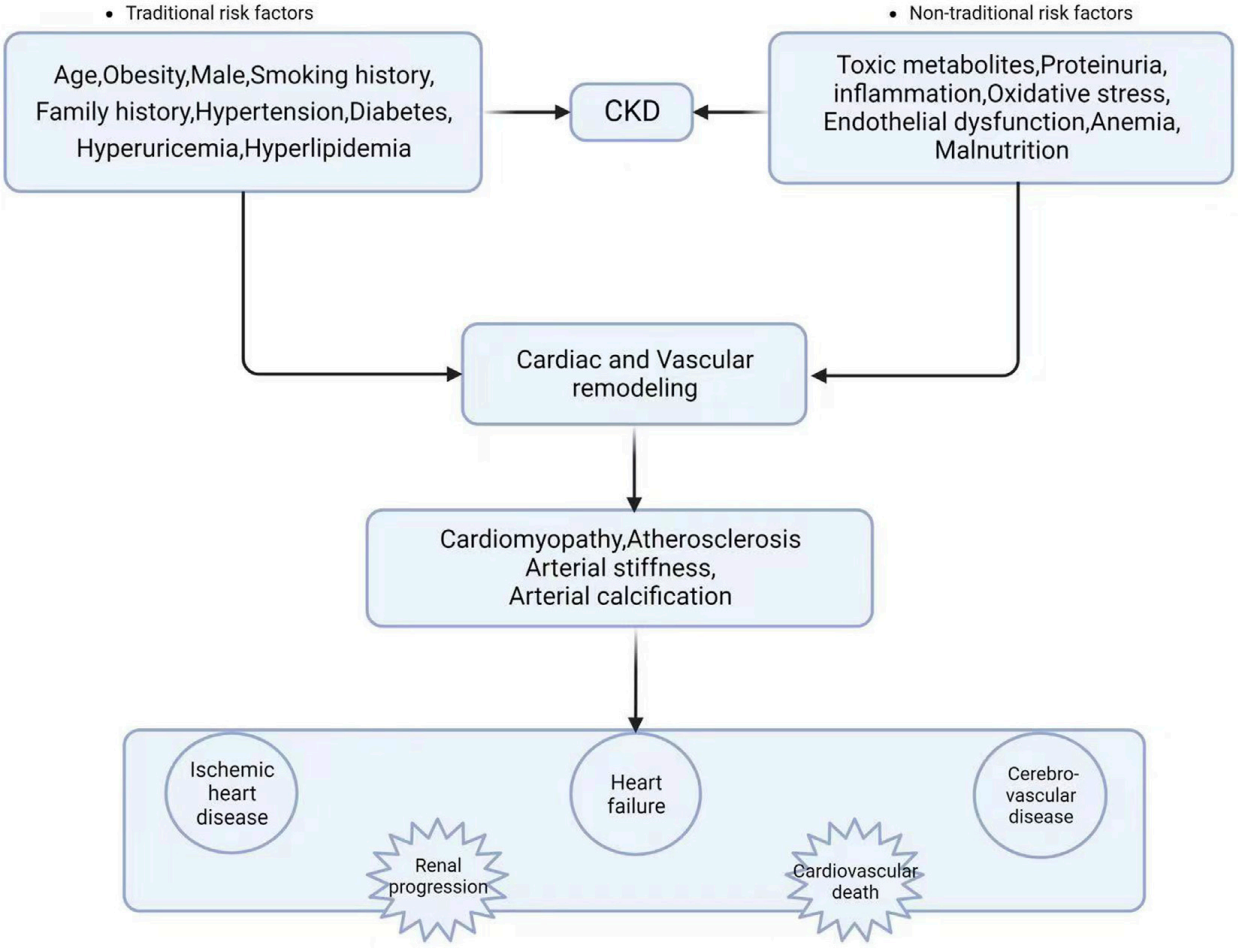
Risk factors associated with CKD combined with CVD and complications (Created with BioRender).

Chronic kidney disease (CKD) is a progressive chronic condition with a high morbidity and death rate, particularly in people with diabetes and high blood pressure. It is a major public health concern that can potentially become the world’s fifth leading cause of death (Kalantar-Zadeh et al., 2021). The risk of cardiovascular disease (CVD) for those with CKD is commonly acknowledged to be increasing, and adverse cardiovascular events continuously increase as the illness worsens. Cardiovascular diseases are a common cause of death in people with chronic kidney disease (CKD) and those with end-stage kidney disease (ESKD), accounting for a significant portion of the deaths in the dialysis community, accounting for more than half of all documented cases. The United States Renal Data System (USRDS) determined in 2020 that the burden imposed by cardiovascular disease (CVD) was much greater on those with chronic kidney disease (CKD) than on those without this illness (Matsushita et al., 2022). CVD was more frequent in advanced CKD patients, with a prevalence of 75.3% in CKD stage 4–5 patients, compared to 66.6% and 63.4% in CKD stage 3 patients and CKD stage 1–2 patients, respectively. The incidence of CVD was 37.5% greater in patients without CKD than in persons with CKD. A recent CCDFRS study (Wang et al., 2023) found that the prevalence of CKD in Chinese adults was 8.2%, with CVD occurring at a rate of 20.6%. CVD mortality has increased in ESKD patients, accounting for 51% of known fatalities among ESKD patients from 2011 to 2013 and 53% and 55% of known deaths among peritoneal dialysis and hemodialysis patients, respectively, in 2018 (Doshi and Wish, 2022). While it is widely assumed that traditional risk factors for cardiovascular disease, such as hypertension, advanced age, dyslipidemia, and diabetes mellitus, are frequently observed in people with chronic kidney disease (CKD), it is important to note that there are additional, unconventional risk factors that are unique to CKD patients. Figure 1 (Sarnak et al., 2003) shows that these “nontraditional” factors include malnutrition, volume overload, anaemia, inflammation, and oxidative stress. Cardiovascular issues such as cardiomyopathy, left ventricular hypertrophy, vascular calcification, and atherosclerosis are more common in CKD patients due to impaired renal function (Sarnak et al., 2003). As a result, CVD increases the risk of morbidity and mortality in CKD patients and is the major cause of death (Lim et al., 2021). Furthermore, the chance of chronic kidney disease (CKD) advancing to end-stage kidney disease (ESKD) increases in tandem with the severity of hypertension. Specifically, CKD patients with mean arterial pressures of 180/100 mmHg had a 15-fold higher risk of developing ESKD than CKD patients with normal blood pressure values (Lim et al., 2021). Troponin and brain natriuretic peptide (BNP), two of the most often utilised indicators in this field, remain increased in CKD patients, perhaps due to decreased renal clearance (Mirna et al., 2020). As a result, their clinical utility in CKD patients is limited. As a result, novel biomarkers are particularly needed to improve the diagnosis and risk stratification of various disease entities for proactive prevention, early detection, prompt intervention, and to reduce the financial burden on patients.

Serum microRNAs are highly stable in the circulatory system and are excellent diagnostic indicators in various diseases (Carracedo et al., 2020). A relationship between miRNA levels and CKD has been discovered in cellular and animal models. Numerous studies have shown that miRNAs have the potential to be indispensable in the area of CKD, and changed miRNA expression has been linked to the initiation and progression of numerous pathological processes, including diabetic nephropathy, renal cancer, and kidney damage. Moreover, because of their intrinsic stability and varied amounts inside both strong and damaged physiological tissues, circulating miRNAs in the circulation play an important role in maintaining the balance of cardiovascular dynamics. Numerous studies have demonstrated that miRNAs can operate as important regulators in CKD-associated CVD signalling pathways, and they are also thought to be biomarkers for the diagnosis and prognosis of a range of illnesses, notably CKD-associated CVDs (Carracedo et al., 2020).

## miRNAs——the core of gene regulation

**FIGURE 2:**
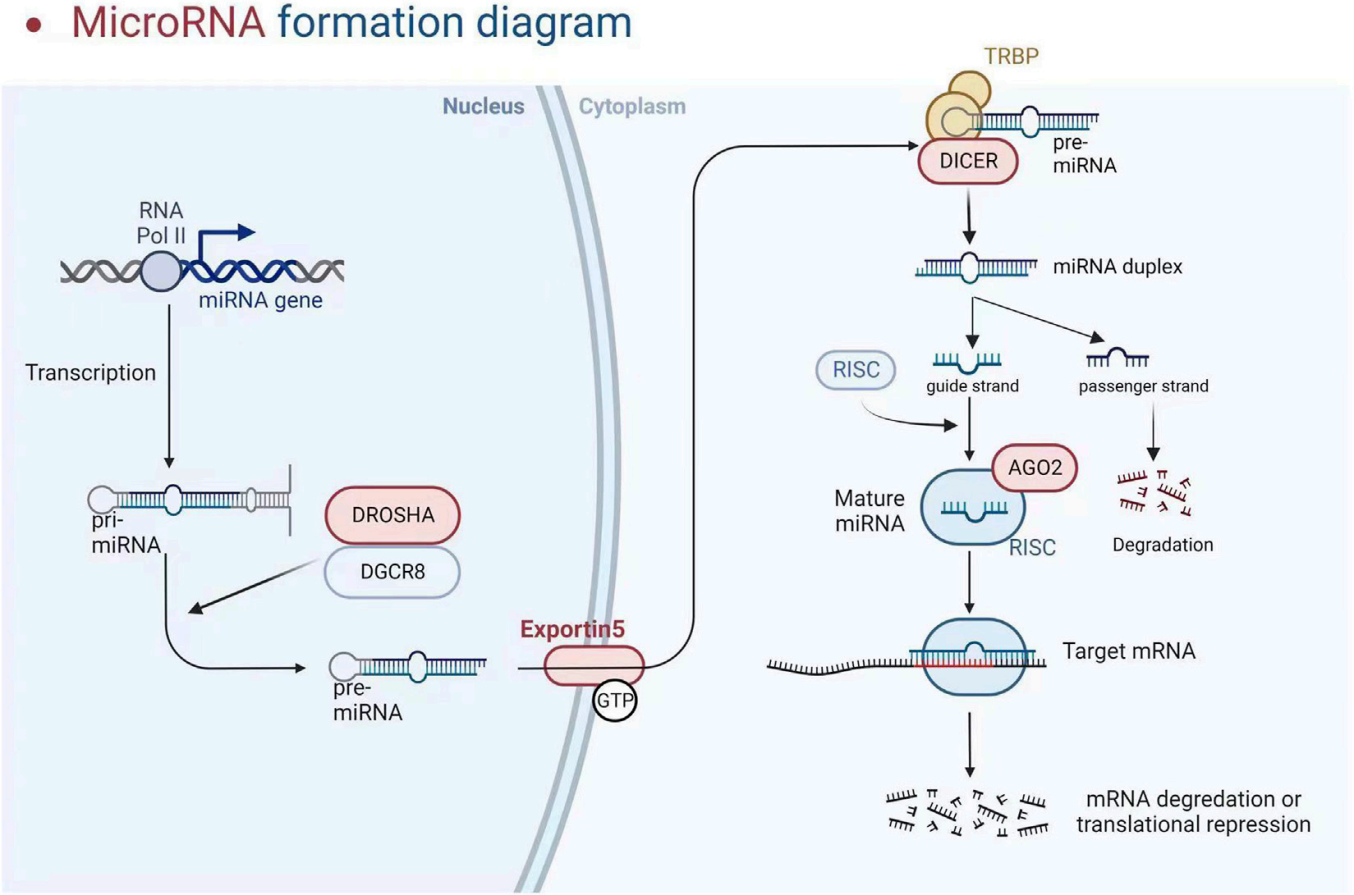
MicroRNA formation diagram (Created with BioRender).

The participation of a recently discovered category of particles known as short non-coding RNAs (200 nucleotides, which includes miRNA, snoRNA, and piRNA) or long non-coding RNAs (lncRNA and circular RNA, >200 nucleotides) is required for gene regulation (Papaioannou et al., 2014; Metzinger-Le Meuth and Metzinger, 2019). We will look at miRNAs in this investigation. These particles are endogenous non-coding RNAs acting as negative gene expression regulators by degrading or trans-suppressing their target mRNAs (Metzinger-Le Meuth et al., 2019). RNA polymerase II converts the miRNA into a longer RNA product (called Pri-miRNA), which is subsequently cleaved in the nucleus by RNase III (Drosha) and DiGeorge syndrome critical region 8 (DGCR8), resulting in miRNA maturation. The length of the produced pre-miRNA hairpin is around 60–70 nt. The export protein transfers it to the cytoplasm, cleaving it into a double-stranded miRNA/miRNA duplex (about 22 bp) by Dicer RNase III. Finally, one of the threads becomes tightly intertwined inside the RISC assembly, known as the RNA-induced silencing complex, allowing it to be transported to the appropriate messenger RNA to inhibit genetic expression. Concurrently, the second thread disintegrates quickly or produces a unique miRNA with clear, albeit variable, biological repercussions (see Figure 2) (Metzinger-Le Meuth and Metzinger, 2019). Although the seed region of mature miRNAs has consistent sequences, some mismatches are common outside this region, allowing a single miRNA to regulate the expression of multiple target genes by repressing target mRNA translation or promoting their degradation, thereby affecting many horizontal developmental pathways (Metzinger-Le Meuth and Metzinger, 2019). MiRNAs can interfere with the start and elongation of particular proteins during translation, reducing their synthesis. They can also isolate and destroy particular mRNAs in cytoplasmic processing bodies. Additionally, targeting promoter regions has been linked to transcriptional gene silencing (Franczyk et al., 2022). The presence of perfect or incomplete complementarity between miRNAs and their target mRNAs allows for regulating several genes. MiRNA-associated gene expression has permanently activated signalling pathways, facilitated cellular phenotypic change in pathological conditions, and accelerated disease progression (Mirna et al., 2020). Long non-coding RNAs (lncRNAs) can improve the functional activity of certain miRNAs by working through “sponge-like” effects or chromatin remodelling. Several studies have discovered that the interaction between these two non-coding RNAs has a role in the genesis and progression of various illnesses (Rong et al., 2017; Metzinger-Le Meuth et al., 2019). MiRNAs are now being studied as predictive biomarkers for illness diagnosis, severity determination, and progression tracking. Several miRNAs are abundantly expressed in the kidney and may play important roles in regulating CKD-related cardiovascular problems. In CKD, paradoxical miRNA expression has an impact on various biological processes and downstream genes that are important for disease initiation or progression.

## miRNAs and CKD-related cardiovascular complications

### miRNAs and vascular calcification

Vascular calcification (VC) is highly prevalent in individuals with chronic kidney disease (CKD) and is strongly linked to major cardiovascular events with devastating consequences. The key underlying process of vascular calcification involves the transformation of vascular smooth muscle cells (VSMCs) into osteoblast-like cells, particularly in the presence of elevated levels of both phosphorus (hyperphosphatemia) and calcium (hypercalcemia). Additionally, the production of matrix vesicles plays a crucial role in facilitating the deposition of calcium and phosphate within the walls of blood vessels (Jansen et al., 2009; Shanahan et al., 2011). Vascular calcification can occur in the vessel’s middle and inner layers, and both calcifications can occur in CKD patients. However, calcification in the intermediate layer is more characteristic of CKD. Initially, vascular calcification was reported in individuals with stage 2 CKD. Vascular calcification affects around 25% of CKD stage 3–4 patients and up to 50%–80% of patients on maintenance hemodialysis. VC accelerates the degradation of renal function. Mineral metabolism disorders, hyperphosphatemia, and excessive use of calcium-binding drugs in the setting of uremia, in addition to established risk factors such as hypertension, dyslipidemia, and inflammation, have emerged as substantial risk factors for VC in CKD. VSMCs are also required for vascular calcification in CKD. Previously, it was believed that vascular calcification (VC) associated with chronic kidney disease (CKD) was a passive phenomenon, characterized by the accumulation of hydroxyapatite within the inner layer of arterial walls due to alterations in calcium and phosphorus metabolism. This process consequently leads to heightened arterial stiffness. Contrary to previous beliefs, recent studies have unveiled vascular calcification (VC) as an active and regulated biological process, resembling the intricate mechanisms involved in bone formation. This process entails the transformation of vascular smooth muscle cells (VSMCs) into osteoblast-like cells, accompanied by the implementation of a cellular program that promotes the deposition of bone matrix within the blood vessels (Leopold, 2015). Under normal circumstances, contractile vascular smooth muscle cells (VSMCs) exhibit a slow rate of proliferation. However, in the presence of mineral dysregulation within a uremic environment, these cells can undergo a phenotypic transformation into an active synthetic phenotype. This transformation leads to an accelerated proliferation rate and enhanced secretory capabilities of the VSMCs (Durham et al., 2018). The initiation of medial calcification has been attributed to the phenotypic transition of VSMCs, which is subsequently accompanied by the degradation of elastin (Disthabanchong and Srisuwarn, 2019). This process involves the downregulation of mineralization inhibitors and the upregulation of molecules that promote calcification. Additionally, the absence of smooth muscle cell indicators (such as SM22a and SMa-actin) and the presence of acquired osteogenic markers [including alkaline phosphatase (ALP), Runx2, and bone morphogenetic protein-2 (BMP-2)] contribute to this phenomenon (Liu and Zhang, 2021). Recent investigations have shed light on the complex and multifaceted progression of vascular calcification. These studies have revealed the presence of inhibitory mechanisms such as fetuin-A, klotho, and the vitamin K-dependent matrix Gla protein (MGP) and Gla-rich protein (GRP). These findings have enhanced our understanding of the intricate processes involved in preventing vascular calcification (Schurgers et al., 2013; Viegas et al., 2019). The detrimental consequences of the pathological condition of CKD, which is distinguished by the occurrence of hypercalcemia, hyperphosphatemia, hyperthyroidism, oxidative stress, inflammation, α Klotho deficiency, and the buildup of uremic toxins such as indole sulfate (is) and advanced glycosylation end products (AGEs) (Zhang et al., 2021). In recent years, miRNA has garnered significant attention as a crucial intercellular signalling molecule and an emerging participant in the field of vascular calcification. It is recognized for its capacity to regulate various processes involved in the development of vascular calcification, thereby serving as an information transporter that promotes this pathological condition.

Runx2, a transcription factor essential for osteoblast and chondrocyte maturation, is involved in VSMC calcification. Runx2 expression is low under normal settings but drastically increased in calcified atherosclerotic plaques, showing that Runx2 may play a role in vascular calcification. Several studies have revealed that miRNAs promote osteogenesis by affecting Runx2 expression directly or indirectly. Overexpression of miR-133a suppresses cytosolic calcification, resulting in the downregulation of RUNX2 and OCN, according to Li et al. (2022) MiR-133a, via targeting the RUNX2 gene, can directly control cytosolic calcification and play a role in the aetiology of end-stage renal disease. MiR-29a and miR-125b target Runx2 indirectly via co-repressors or co-activators, which may result in increased or reduced control of osteoblast differentiation (Narayanan et al., 2019). MiR-125b, according to Chao et al. (2017), might be used as a predictive biomarker for VC. MiR-125b levels in the blood were shown to be inversely related to the severity of VC, meaning that miR-125b may be used as a risk factor for VC in uremia. Endogenous miR-125b inhibition improved osteogenic transdifferentiation, alkaline phosphatase activity, and matrix mineralisation. According to expression data, miR-125 b targets the osteoblast transcription factor SP7 (osterix). ApoE knockout mice corroborated this (Goettsch et al., 2011). In an *in vitro* biomineralisation model, a discernible difference in osteocalcin expression was observed between VSMC that underwent transfection with miR-125b and the control VSMC (Chao et al., 2017). The corroboration of these findings can be ascertained by the observation that a decrease in the circulation of miR-125b among patients suffering from chronic kidney disease is intricately linked to a significant deterioration in renal function (Chen et al., 2013). Significant correlations have been found between the levels of miR-125b and the severity of vascular calcification (VC), as well as the levels of fetuin-A and the mediators of mineral bone disease, osteoprotegerin, and FGF-23 (Chao et al., 2017; Chao et al., 2019). High blood osteoprotegerin levels and low serum miR-125b levels enhanced VC risk assessment in one investigation (Chao et al., 2019). Hyperparathyroidism manifests itself in early CKD and can be noticed in dialysis patients, where elevated parathyroid hormone levels are a symptom of bone and mineral abnormalities (Lee et al., 2019). Recently, the role of microRNAs in parathyroid function has been studied. Shilo et al. (2017) discovered that miR148 and let-7 control parathyroid hormone production in secondary hyperparathyroidism, with let-7 members inhibiting PTH secretion and miR-148 members promoting it. Increased parathyroid let-7 expression and miR-148 members in CKD may lead to SHP formation. Future research may reveal miRNAs as new therapeutic targets for SHP management. It is uncertain what causes the present study’s direct link between miRNAs and parathyroid hormone levels. As a result, additional investigation into this relationship is required. In a rat model of CKD, miR-302b levels dropped in chronic renal failure (CRF) animals. Upregulation of miR-302b, on the other hand, may increase calcium and phosphorus metabolic activity and prevent the development of ectopic VC, thereby alleviating the condition of CRF that may be related to the BMP-2/Runx2/Osterix signalling pathway, thereby providing a novel concept for identifying effective CKD treatments (Sun et al., 2018). Exosomes generated from mesenchymal stem cells in the bone marrow, also known as BMSC-Exo, have significantly reduced calcification caused by hyperphosphatemia inside vascular smooth muscle cells (VMSCs). This finding implies that BMSC-Exo has enormous potential in treating chronic kidney disease-associated vascular calcification (CKD-VC). However, the particular mechanical foundations of this event remain unknown and deserve additional exploration. A scientific study used hyperphosphate-stimulated human aortic smooth muscle cells (HA-VSMCs) in conjunction with a 5/6 complete nephrectomy (SNx) rat model to investigate the complicated workings of BMSC-Exo. This study sought to determine the underlying mechanism of action of BMSC-Exo using both human and animal experimental models. The current study discovered that BMSC-Exo effectively inhibits the progression of apoptosis and calcification *in vivo* and *in vitro* via the mediating influence of microRNA-381-3p (miR-381-3p). In addition, it has been unequivocally demonstrated that miR-381-3p effectively inhibits the production of nuclear factor 5 (NFAT5) in vibrant T cells via its astute binding to the 3′ untranslated region. Furthermore, the presence of significant arterial calcification among dialysis patients has been found to have a negative correlation with the level of miR-381-3p while having a positive correlation with the expression of NFAT5. This study concluded that BMSC-Exo had anti-calcification and anti-apoptotic effects in CKD by providing closed miR-381-3p and directly targeting NFAT5 mRNA (Liu et al., 2022). In a built-rat CRF model, overexpression of miR-93 in the aorta of CRF rats significantly reduced the production of OPN, OCN, RUNX2, and BMP-2. MiR-93 inhibited aortic calcification by delaying VSMC osteogenic differentiation. MiR-93 may reduce Wnt/- signalling pathway activity and VC-related gene expression to enhance renal function in CRF rats and relieve vascular calcification in CRF, establishing the framework for VC-targeted treatment in CRF (Peng et al., 2022).

SULF1, which encodes heparan sulphate lyase 1, is widely distributed in tissues and has the ability to influence Wnt, FGF, and BMP pathways by modifying extracellular proteoglycans. The role of SULF1 in vascular smooth muscle cell (VSMC) physiology cannot be overstated. Evidence suggests that increased SULF1 expression in VSMC causes a decrease in adhesive properties while simultaneously increasing the propensity for apoptosis, migration, and chemotaxis (Sala-Newby et al., 2005). Moreover, SULF1 stimulation of vascular tissue Wnt signalling has been established (Sahota and Dhoot, 2009), while induction of the Wnt/-catenin pathway promotes the formation of vascular calcification (Nie et al., 2019). The incorporation of SULF1 is postulated as a potential modulator in the regulation of angiotensin II receptor expression and consequently, it exhibits the ability to augment osteogenic differentiation, as alluded to by previous studies (Cha et al., 2018; Matsushita et al., 2015). MiR-378a-3p, wi-th its interaction with BMP2 and ERK2, purportedly assumes a pivotal function in terms of skeletogenesis and calcification. Notably, higher BMP2 levels have been regularly found in the setting of calcified aneurysms, making it a widely recognised marker. Surprisingly, lowering BMP2 levels has been shown to reduce the severity of vascular calcification (VC). Activation of ERK1/2, an intracellular signalling pathway, has been linked to the transdifferentiation of vascular smooth muscle cells (VSMCs) into osteoblasts. A notable corollary could be the increased potential for vascular calcification (VC) achieved by using compounds that inhibit ERK1/2 expression. Another potential target of miR-378a-3p, Ca2+/calmodulin-dependent protein kinase II (CAMKK2), has been shown to induce stem cell differentiation in the osteoblast lineage (Chao et al., 2021). Chao et al. (2021) discovered that serum miR-378a-3p, when combined with their matched gene SULF1, was independently linked with serum VC and VC severity in sera from patients with nondialysis CKD and dialysis-dependent ESRD, outperforming clinical characteristics alone or target proteins alone. Finally, miRNA-target gene pairings functionally govern the pathogenic process of VC and may be therapeutic targets. The findings of this study constitute a significant step forward in identifying uremic individuals at high risk using molecular regulation-based (miRNA-mRNA) paired biomarkers.

IS, the representative uremic toxin, can induce osteogenic differentiation in vascular smooth muscle cells (VSMCs) by enhancing oxidative stress levels, changes in methylation patterns, and changes in the expression of microRNAs (miRNAs) specifically involved in osteogenic differentiation. These molecular mechanisms result in a condition known as vascular calcification (VC), which has a strong association with the presence of VC in patients with chronic kidney disease (CKD) (He et al., 2020). Matrix GLa protein (MGP) is a calcification inhibitor that plays an important role in VC. MGP was reduced in calcified arteries *in vitro* by IS through activation of ROS/NF-B signalling to suppress MGP expression in HASMCs in parallel with osteogenic differentiation, and further investigation revealed that IS induced NF-B-responsive microRNA (miR)-155. The rise in miR-155-5p mimic overexpression enhanced the decrease in MGP generated by IS and osteogenic cell differentiation. However, these states were alleviated by silencing miR-155-5p. IS induces a phenotypic change in HASMCs by reducing MGP expression via ROS/NF-B/miR-155-5p signalling, shedding light on the pathophysiology of IS-induced VC (He et al., 2020). IS was found to have a dual effect on human aortic smooth muscle cells (HASMCs), causing a decrease in miR-29b expression while increasing calcification. MiR-29b mimics strongly suppressed IS-induced expression of Runt-related transcription factor 2 and Osteopontin, but miR-29b anti-miR was significantly enhanced. In comparison to control participants, patients with end-stage renal disease had higher levels of Wnt7b/-catenin expression in the radial arteries. Additionally, it was observed that the influence of IS resulted in an intensified manifestation of Wnt7b/β-catenin in human aortic smooth muscle cells (HASMCs), whereby this augmentation occurred within a mere duration of 3 days following the initiation of stimulation. Moreover, miR-29b mimics inhibited Wnt7b/β-catenin protein expression in HASMCs while miR-29b anti-mir enhances it, demonstrating that miR-29b adversely controls Wnt7b/β-catenin signalling. The protein known as Dickkopf-1 was shown to have an inhibitory effect on the calcification process in human airway smooth muscle cells (HASMC). This calcification is induced by the presence of anti-miR-29b, a factor of significant influence in the suppression of the Wnt/β-catenin signalling pathway. As a result, the findings of this study suggest that the suppression of miR-29b and the activation of Wnt/β-catenin signalling may emerge as a pivotal mechanism in the occurrence of vascular calcification stimulated by IS in the context of chronic renal disease (Zhang et al., 2018). However, given the intricacy of the process’s regulation and the diversity of miRNAs involved, additional research is needed to find the best candidate miRNAs for targeting (See Table 1).

**TABLE 1:** Comparison of miRNAs in vascular calcification.

miRNAs	Population/Methods	Function	Key-findings
miR-133a Li et al. (2022)	Rat smooth muscle A7r5 cells	MiR-133a is mainly expressed in myocardial and skeletal muscle cells, which regulates their proliferation and differentiation	● miR-133a can indirectly regulate cellular calcification through RUNX2 gene expression. The results of this study provide insight into miR-133a as a molecular target for the diagnosis of vascular calcification in end-stage renal disease
miR-125b Goettsch et al. (2011); Chao et al. (2017)	rat and human aortic vascular smooth muscle cells	miR-125b plays a role in atherosclerotic plaque formation, including inflammation, apoptosis, and angiogenesis	● serum miR-125b levels are associated with VC severity and serve as a novel predictive marker for the risk of uremia-associated calcification progression ● It demonstrate that miR-125b is involved in the osteogenic transdifferentiation of VSMCs, at least in part by targeting SP7, and implicate miRs as a novel link for the common mechanisms of vascular calcification and bone remodeling
miR-148 Shilo et al. (2017)	male Sprague Dawley rats and C57BL/6 mice	miR-148 family has been linked with various neoplastic diseases, and inhibition of miR-148 was reported to downregulate insulin mRNA in pancreatic islet cells	● let-7 members restrict PTH secretion, whereas miR-148 members promote secretion. In CKD, the expression of parathyroid let-7 and the increase in miR-148 members may contribute to the development of SHP. Future studies may identify miRNAs as new therapeutic targets for the management of SHP, a common complication of CKD and a source of morbidity and mortality in these patients
miR-302b (Sun et al., 2018)	male clean-grade Sprague-Dawley rats	miR-302 could promote the activation of BMP signaling pathway in undifferentiated human embryonic stem cells	● miR-302b was shown to be decreased in rats with CRF, and upregulation of miR-302b may improve calciumphosphorous metabolism and prevent the progression of ectopic VC, thus relieving the conditions of CRF possibly associated with BMP-2/Runx2/Osterix signaling pathway, which provided a new idea for finding effective therapeutic approach to CRF.
miR-381-3p Liu et al. (2022)	Human aortic smooth muscle cells (HAVSMCs) and 5/6 subtotal nephrectomy (SNx) rat	miR-381-3p as a dual suppressor of TNF-induced apoptosis and necroptosis in multiple cancer cells	● BMSC-derived exosomal miR-381- 3p could downregulate NFAT5, therefore alleviate the cellular apoptosis and VC. This study reveals the significant role of miR-381-3p/NFAT5 in regulating apoptosis/Vascular calcification。
miR-93 Peng et al. (2022)	Sprague Dawley (SD) male rats	miR-93 in the development, progression and drug tolerance of multiple tumors, but also pointed out its critical roles in senescence, hyperglycemia and osteoblast calcification	● MiR-93, via inhibiting the activity of Wnt/β-catenin pathway by targeting TCF4, can improve the renal function of CRF rats, thereby mitigating the vascular calcification of CRF.
miR-378a-3p Chao et al. (2021)	rat aortic vascular smooth muscle cells (VSMCs) A7r5 and human aortic smooth muscle cells (ASMCs)	miR-378a-3p exhibits multifaceted effects on angiogenesis, muscle cell proliferation and differentiation, and it promotes myogenesis, myocyte survival and inhibits apoptosis	● After analyzing and screening the probability of incorporating miR-378a-3p and SULF1 into these miRNA/protein biomarkers for the measurement of uremic VC, it is possible that significant improvements could be made in how we diagnose uremic VC.We believe that a combined biomarker set including miRNAs and their target proteins may represent a potentially useful approach for identifying patients who are prone to VC or who may experience ESRD patients with worsening VC
miR-155-5p He et al. (2020)	human aortic smooth muscle cells (HASMCs)	MiR-155-5p is a typical NF-κB-responsive miRNA, as a NF-κB-binding site is present in its promoter, which has marked effects on endothelial dysfunction, vascular inflammation, and VSMC phenotypic	● IS promotes the HASMCs phenotype switch by suppressing MGP expression via ROS/NF-κB/miR-155-5p signaling and provide a new insight for the pathogenesis of IS-induced VC.
miR-29b Zhang et al. (2018)	human aortic smooth muscle cells (HASMCs)	A feedback loop exists between the miR-29 family and wnt signaling, which contributes to osteoblastic differentiation of MSCs	● miR-29b as an important protective factor of VSMC calcification by repressing wnt/β-catenin signaling, which represents a novel mechanism regulating vascular calcification. This finding may provide a new perspective on pathogenesis and treatment of vascular calcification in chronic kidney disease

### miRNAs and left ventricular hypertrophy

The presence of left ventricular hypertrophy (LVH) is a prevalent characteristic of cardiac alterations observed in individuals with chronic renal illness, contributing to the development of heightened cardiovascular abnormalities (Shimizu and Minamino, 2016). Left ventricular hypertrophy is observed in the first phases of chronic kidney disease (CKD) and is prevalent in around 65% of patients who have not yet initiated dialysis (Parfrey et al., 1990). Hemodynamic overload has been widely recognised as a significant catalyst for the occurrence of left ventricular hypertrophy (LVH) in individuals with chronic kidney disease (CKD) (Zoccali et al., 2017). Additionally, various detrimental factors linked to CKD, including the renin-angiotensin system (RAS), uremic toxins, microinflammatory states, and disturbances in phosphorus metabolism, have shown a strong correlation with LVH (Wang and Shapiro, 2019). Identification of a shared mechanism underlying cardiac hypertrophy generated by these causal variables has the potential to establish a foundation for understanding left ventricular hypertrophy in chronic kidney disease (CKD) and subsequently enhance the efficacy of therapy for CKD patients with cardiovascular problems. MicroRNAs (miRNAs) are a category of brief, non-coding ribonucleic acids (RNAs) that govern the modulation of gene expression subsequent to transcription. MicroRNAs (miRNAs) play a significant role in the development of cardiac hypertrophy and have the potential to serve as mediators in the progression of hypertrophy. The expression of miR-30 is significantly upregulated in cardiac tissues and downregulated in hypertrophic circumstances, indicating its crucial role in cardiac function. The miR-30 family has high levels of expression in cardiac tissues during normal physiological settings (Mcgahon et al., 2013; Ludwig et al., 2016), indicating their significant involvement in heart function. Following the surgical removal of a kidney (nephrectomy), the occurrence of cardiac hypertrophy and inhibition of myofibrillar miR-30 were observed. These findings primarily indicate a potential association between miR-30 inhibition and the advancement of cardiac hypertrophy in chronic kidney disease (CKD). Bao et al. (2021) conducted a study that revealed the inhibition of endogenous miR-30 in cardiomyocytes resulted in the development of pathological cardiac hypertrophy. Additionally, this inhibition augmented the activity of calmodulin phosphatase, leading to increased phosphorylation, and facilitated the translocation of NFATc3 to the nucleus. In addition, the transfection of miR-30 sponge plasmid into cardiomyocytes resulted in the manifestation of cardiomyocyte enlargement and increased intranuclear NFATc3 concentrations. Moreover, cardiomyocyte hypertrophy was inhibited upon treatment with the particular calmodulin inhibitor FK506. Significantly, the mRNAs of Ppp3ca and Nfatc3 are directly regulated by miR-30. In addition, it was shown that miR-30 had a mitigating effect on calcineurin signalling inside the myocardium of rats with chronic kidney disease (CKD) and mice treated with fibroblast growth factor-23 (FGF-23). Thus, it can be inferred that the association between calmodulin/NFATc3 and miR-30 contributes to cardiac hypertrophy. Reducing miR-30 is of utmost importance in developing left ventricular hypertrophy produced by chronic kidney disease. A recent study conducted by Chuppa et al. (2018) demonstrated that the suppression of miR-21 has a protective effect against left ventricular hypertrophy (LVH) and leads to an improvement in left ventricular (LV) function in rats with 5/6 nephrectomy. This beneficial effect was seen to occur through the activation of the peroxisome proliferator-activated receptor alpha (PPAR-α) pathway. Furthermore, there have been reports indicating that the activation of Na+/K+-ATPase signalling results in the suppression of miR-29b expression in the heart, hence promoting elevated collagen production in individuals with chronic kidney disease (Drummond et al., 2016). Furthermore, the downregulation of miR-29b and miR-30c has been implicated in the development of cardiac fibrosis in chronic kidney disease (CKD). These microRNAs play a crucial role in regulating collagen-1a1, matrix metalloproteinase 2, and connective tissue growth factors, all of which are well-established promoters of fibrosis (Panizo et al., 2017). The suppression of miR-208 in the cardiac system has been observed to be linked to cardiac hypertrophy in individuals with chronic kidney disease (CKD) (Prado-Uribe et al., 2013).

The role of miR-212 as a crucial regulator of pathological left ventricular hypertrophy in heart failure generated by pressure overload has been demonstrated through its regulation of the FOXO3/calreticulin/nuclear factor of activated T cells (NFAT) pathway. In a recent study conducted by Sárközy et al. (2019), the researchers investigated the potential role of miR-212 and its associated targets, including FOXO3, extracellular signal-regulated kinase 2 (ERK2), and AMP-activated protein kinase (AMPK), in the pathogenesis of heart failure with preserved ejection fraction (HFpEF) within the context of chronic kidney disease (CKD) patients. Chronic kidney disease (CKD) was experimentally created in male Wistar rats using a surgical procedure known as 5/6 nephrectomy. The echocardiography and histological examinations revealed the presence of left ventricular hypertrophy, fibrosis, maintained systolic function, and diastolic dysfunction in the group with chronic kidney disease (CKD) at the end of 8 and/or 9 weeks, in comparison to the animals who underwent sham surgery. The expression of miR-212 in the left ventricle was considerably upregulated in the group with chronic kidney disease (CKD). Nevertheless, the mRNA and protein levels of FOXO3, AMPK, and ERK2 remained relatively unchanged. The protein kinase B (AKT)/FOXO3 and AKT/mammalian target of rapamycin (mTOR) pathways have been implicated as potential mechanisms involved in the regulation of left ventricular hypertrophy (LVH) induced by pressure overload. It is worth noting that the ratio of phosphorylated-AKT to total AKT was found to be elevated in individuals with chronic kidney disease (CKD), with no significant impact on the phosphorylation of FOXO3 or mTOR. In summary, the cardiac overexpression of miR-212 in chronic kidney disease (CKD) did not have any impact on the downstream targets that have been previously linked to hypertrophy. Therefore, it can be inferred that the molecular processes responsible for the development of left ventricular hypertrophy (LVH) in chronic kidney disease (CKD) are not influenced by FOXO3, ERK1/2, AMPK, and AKT/mTOR-mediated pathways. This implies that this particular kind of LVH possesses distinct characteristics. This work is the initial investigation documenting the co-occurrence of left ventricular hypertrophy (LVH) and fibrosis with discernible overexpression of miR-212 in the left ventricle among individuals with chronic kidney disease (CKD). Further inquiry is imperative in order to ascertain whether the upregulation of miR-212 in cardiac tissue serves as a driving force or a consequence of left ventricular hypertrophy associated with chronic kidney disease.

Li et al. (2020) conducted a study on ventricular hypertrophy and demonstrated that circRNA_000203 exhibited upregulation in cardiac hypertrophy. Moreover, the researchers observed that circRNA_000203 contributed to the augmentation of heart hypertrophic growth both *in vivo* and *in vitro*. Circular RNA 000203 has the ability to sequester miR-26b-5p and miR-140-3p, resulting in an upregulation of GATA4 expression, hence facilitating the development of cardiac hypertrophy. The study also determined that the activation of the NF-κB signalling pathway had a role in the increased expression of circRNA_000203 during cardiac hypertrophy. The microRNA known as miR-133a has putative regulatory functions in the context of cardiac hypertrophy. The activation of hypertrophic signalling through the involvement of NFAT (nuclear factor of activated T cells) is a crucial regulatory response to hypertrophic stimuli. The transcription factor NFATc4, which is related to hypertrophy, is a target of negative regulation by miR-133a. Moreover, the *in vitro* upregulation of miR-133 or miR-1 showed inhibitory effects on cardiac hypertrophy. On the contrary, the induction of hypertrophy by the use of a “decoy” sequence to block miR-133 exhibited a greater degree of prominence compared to the hypertrophy caused by conventional inducers. The induction of heart hypertrophy is shown as a persistent and significant outcome when miR-133 is inhibited *in vivo* with a single transfection of antigametophytes. The particular targets of miR-133 have been identified as rhoA, a protein responsible for GDP-GTP exchange and regulation of cardiac hypertrophy; Cdc42, a signalling kinase involved in hypertrophy; and Nelf-A/WHSC2, a nuclear factor implicated in cardiogenesis (Care et al., 2007). In addition, the presence of myocardial ischemia, along with the measurement of circulating miR-133a, has the potential to serve as a biomarker for the prediction of cardiac hypertrophy in individuals undergoing chronic hemodialysis and those who have had valve replacement due to aortic stenosis (Xiao et al., 2019). In a murine model of ventricular hypertrophy produced by Ang II, the exosome miR-21 generated from cardiac fibroblasts has a hypertrophy-promoting influence by selectively targeting the Z-lineage-associated proteins SORBS2 and PDLIM5 (Fang et al., 2022). In a similar vein, a recent study has demonstrated that the expression of miR-27a is increased in hearts affected by infarction, leading to the promotion of hypertrophic gene expression. This effect is achieved through the targeting of PDLIM5, which in turn facilitates the process of cardiomyocyte hypertrophy (Tian et al., 2020). The upregulation of miR-217 expression was seen in both transverse aortic constriction (TAC) mice models and people with heart failure (HF) (Nie et al., 2018). According to a recent study, the process of converting fibroblasts into induced pluripotent stem cells (iPSCs) was shown to have a mitigating effect on cardiac hypertrophy. This effect was achieved by the reduction of exosomal miR-22, as indicated by the study (Huang et al., 2013).

Paterson et al. (2019) conducted a compelling study to examine the gender-specific impact of miR-146b-5p on cardiology within a rat model of chronic kidney disease (CKD). Following the administration of 5/6 Nx, female miR-146b−/− rats did not exhibit observable cardiac hypertrophy as compared to their wild-type counterparts. However, it is worth noting that aberrant renal function was seen in the former group. The expression of this trait was partially diminished with the use of a preventive ovariectomy procedure. On the other hand, the removal of miR-146b-5p did not result in any significant impact on the hypertrophic response in males. Instead, men displayed noticeable left ventricular hypertrophy and cardiac chamber dilatation after undergoing 5/6 Nx when compared to miR-146b−/− males but not when compared to wild-type males. The results of sophisticated computational calculations demonstrated that the primary target of miR-146b-5p activity was the downregulation of TGFB1 protein production in the TGF-B pathway, together with the suppression of waveform protein (Vim) and e-calmodulin (Cdh1). The administration of β-estrogen resulted in a decrease in the expression of Vim induced by TGF-β. Conversely, treatment with pre-miR-146b significantly reduced collagen expression in cells exposed to both TGF-β and β-estrogen but not in cells incubated solely with TGF-β. These findings suggest that the effects of miR-146b-5p are influenced by sex hormones. Therefore, it may be inferred that miR-146-5p in women with wild-type (WT) genes may enhance the beneficial impacts of oestrogen. This might potentially elucidate the reason for the disruption of gonadotropin signalling and the deterioration of chronic kidney disease (CKD) when miR-146b-5p is inhibited. Further investigation is required to explore the potential involvement of additional miRNA families in regulating hormonal responses to cardiac pathology during chronic kidney disease (CKD) (D'Agostino et al., 2023). The aforementioned studies not only provide insights into a hitherto unidentified mechanism of left ventricular hypertrophy (LVH) production in chronic kidney disease (CKD) but also propose prospective therapeutic targets for managing cardiac hypertrophy in CKD patients (refer to Table 2).

**TABLE 2:** Comparison of miRNAs in Ventricular Hypertrophy.

miRNAs	Population/Methods	Function	Key-findings
miR-30 Bao et al. (2021)	male Sprague-Dawley rats	miR-30 regulates autophagy, apoptosis and oxidative stress	● MiR-30 suppression may result in cardiac hypertrophy via calcineurin/NFATc3 activation in LVH.
miR-21-5p Chuppa et al. (2018)	Male Sprague Dawley rats	miR-21 has a role in acute and chronic pathology in several organs. Short term cardiac upregulation of miR-21 is protective in ischemia/reperfusion injury, while chronic upregulation has been associated with pathologic changes.miR-21 suppression has also been reported to be renoprotective in models of acute renal injury/fibrosis and Alport nephropathy	● Inhibition of miR-21-5p protects 5/6 nephrectomized rats from developing left ventricular hypertrophy and improves left ventricular function ● MiR-21-5p inhibition altered gene expression of the left ventricular peroxisome proliferator-activated receptor α (PPAR α) regulatory pathway. PPAR α, a miR-21-5p target, is the predominant PPAR isoform in the heart and plays an important role in the regulation of fatty acid metabolism.Therapeutic administration of a low-dose PPAR α agonist (clofibrate) to 5/6 nephrectomized rats improved cardiac function and prevented left ventricular dilatation
miR-29b and miR-30c Panizo et al. (2017)	male Wistar rats	miR-29 targets genes encoding collagen types I and IV and MMP-2, among others. Downregulation of miR-29 by TGF-b1/Smad3 is part of the mechanism by which this signalling pathway induces renal fibrosis. In addition, the loss of miR-30 in the course of LV hypertrophy intensifies pro-fibrotic signalling, in part through increases in CTGF levels	● Vitamin D receptor activators (VDRAs), particularly paricalcitol, attenuated cardiac fibrosis acting on COL1A1, MMP-2 and CTGF expression, partly through regulation of miR-29b and miR-30c
miR-208 Prado-Uribe et al. (2013)	Male Sprague Dawley rats	microRNA-208 (mir-208), is selectively expressed in myocardial tissue and is involved in the control of heart remodeling because it regulates the expression of b-MHC and myocardial fibrosis in response to various stimuli	● Decreased plasma level of thyroid hormones or sensitivity at tissue level observed in chronic kidney disease induced by 5/6Nx has an important effect in heart remodeling processes, some of it related or mediated by mir-208 and TGF-b expression in the heart
miR-212 Sárközy et al. (2019)	adult male Wistar rats	miR-212 overexpression plays a role in the development of LVH and heart failure through fetal gene reprogramming in the human heart. In addition, the prohypertrophic potential of miR-212 was confirmed in primary neonatal rat cardiomyocytes	● cardiac overexpression of miR-212 in CKD failed to affect its previously implicated hypertrophy-associated downstream targets. thus, the molecular mechanism of the development of LVH in CKD seems to be independent of the FOXO3, ERK1/2, AMPK, and AKT/mTOR-mediated pathways indicating unique features in this form of LVH.
miR-133a Care et al. (2007)	10–12 week old C57BL/6 female mice (Harlan)	miR-133a inhibits angiogenesis, apoptosis, fibrosis, hypertrophy, and inflammation while promoting therapeutic cardiac remodeling	● MiR-133 has a critical role in determining cardiomyocyte hypertrophy
			● Overexpression of miR-133 or miR-1 *in vitro* inhibited cardiac hypertrophy. In contrast, induction of hypertrophy by miR-133 was inhibited by a “decoy” sequence, which was more pronounced than after stimulation with conventional hypertrophy inducers
			● We identified specific targets of miR-133: RhoA, a GDP-GTP exchange protein that regulates cardiac hypertrophy; Cdc42, a signaling kinase implicated in hypertrophy; and Nelf-A/WHSC2, a nuclear factor involved in cardiogenesis
			● MiR-133 and possibly miR-1 are key regulators of cardiac hypertrophy, suggesting their therapeutic application in heart disease
miR-27a Tian et al. (2020)	male Sprague-Dawley rats	MicroRNA-27a (miRNA-27a) is the major microRNA contained in cardiac fibroblast-derived EVs and contributes to oxidative stress as well as expression of hypertrophic genes in cardiomyocytes	● Secretion of miRNA27a*-rich EVs by cardiac fibroblasts may serve as a paracrine signaling mediator of cardiac hypertrophy with potential as a new therapeutic target.
miR-217 Nie et al. (2018)	Seventeen heart samples were collected from cardiac transplant patients with CHF, and nine heart samples were from accident victims. Male C57BL/6 mice	MiR-217 plays multiple roles in physiological and pathological processes. For example, mir-217 promotes inflammation and fibrosis through the SIRT1/HIF-1a signaling pathway in high glucose-cultured rat glomerular mesangial cells. miR-217 mediates the protective effects of dopamine D2 receptors against fibrosis by targeting Wnt5a in human renal proximal tubule cells. Overexpression of transforming growth factor b1 (TGF-b1) triggers dysregulation of the miR-217-SIRT1 pathway, which then promotes the epithelial-mesenchymal transition process	● The findings reveal that miR-217 is highly expressed in the hearts of CHF patients and aggravate pressure overload-induced cardiac hypertrophy and dysfunction by suppressing PTEN expression. Cardiomyocyte-derived miR-217-containing exosomes induce fibroblast proliferation and may promote cardiac fibrosis (Figure 7). These findings suggest that miR-217 plays important roles in cardiac hypertrophy and dysfunction, providing therapeutic targets for heart failure
miR-22 Huang et al. (2013)	This study established mouse models of global and cardiac-specific miR-22 deletion	miRNA-22 (miR-22) was previously reported as a tumorsuppressive miRNA that induces cellular senescence in cancer cell lines. miR-22 was shown to repress hypertrophy in cultured cardiomyocytes.miR-22 was required for the heart to adapt to pressure overload–induced cardiac hypertrophy	● miR-22 as a critical regulator of cardiomyocyte hypertrophy and cardiac remodeling
miR-146b-5p Paterson et al. (2019)	miR-146b-5p knockout rats	miR-146b-5p, is an important mediator in renal cardiac pathophysiology. Increased abundance of miR-146b-5p has been reported in clinical studies in renal pathology and experimental disease models; including hypertension, AKI, renal fibrosis and CKD.	● MiR-146b rats exhibited functional knockout of miR-146b-5p in both kidney and heart
			● Although 5/6 nephrectomy resulted in tissue hypertrophy, miR-146b−/− female rats showed exacerbated renal hypertrophy
			● In addition, miR-146b female rats exhibited significantly elevated renal fibrosis and marked renal insufficiency, but lower blood pressure and less pronounced cardiac remodeling. These phenotypic differences were not demonstrated in miR-146b−/− male rats
			● Ovariectomy improved renal pathology and eliminated genotypic differences

### miRNAs and endothelial dysfunction

Endothelial dysfunction, a pathological condition affecting the inner lining of blood vessels, is a primary contributor to the development of chronic kidney disease and significantly increases the likelihood of cardiovascular complications (Roumeliotis et al., 2020). Several recently discovered endothelium damage indicators can be utilised to assess this process in CKD (Glorieux et al., 2021). Syndecan-1 (Sdc-1) belongs to the transmembrane heparan sulfate glycoprotein family, serving as a reservoir for growth factors and chemokines that play crucial roles in numerous cellular activities (De Luca et al., 2010). VCAM-1 (vascular cell adhesion molecule) is a glycosylated protein located on the surface of endothelial cells. Its primary function is to facilitate the recruitment of macrophages to the renal vasculature in response to vascular injury (Chow et al., 2005). During tissue remodeling, matrix metalloproteinase-7 (MMP-7) plays a crucial role in the breakdown of the extracellular matrix (Bradshaw, 2009). MMP-7 is found to be increased in individuals suffering from chronic renal illness, positioning it as a significant biomarker for assessing endothelial dysfunction (Liu et al., 2020). Angiopoietin-2 (ANGPT2) influences endothelial cell death, migration, and proliferation via altering cell-matrix interaction (David et al., 2010). The buildup of uremic toxins in the blood is another characteristic of chronic renal failure (Barreto et al., 2009). On the basis of their molecular weights and protein-binding capacity, uremic toxins are classified as (a) water-soluble small molecular compounds (e.g., inorganic phosphates), (b) medium molecular compounds, and (c) protein-bound compounds [e.g., indole sulphate (IS)]. Uremic toxins can induce endothelial dysfunction in patients with chronic kidney disease (CKD). Indeed, the endothelium monolayer structure is altered in chronic kidney disease patients, and studies have demonstrated that uremic toxins cause cell-cell junction loss and increased permeability. Uremic toxins induce oxidative stress within these cells, triggering the activation of signaling pathways including the aryl hydrocarbon receptor (AhR), nuclear factor-B (NF-B), and mitogen-activated protein kinase (MAPK). When these pathways are activated, pro-inflammatory proteins (e.g., monocyte chemotactic protein-1, E-selectin) and pro-thrombotic proteins (e.g., tissue factor) are overexpressed. Uremic toxins have been found to induce the production of endothelial cell microparticles (EMPs), leading to the activation and subsequent dysfunction of various cellular components. These toxins also disrupt the expression of microRNAs, critical regulators of cellular functions (Cunha et al., 2020). As a result, uremic toxins and the pathways they control may be viable therapeutic targets for improving CKD therapy.

According to Cunha et al., individuals with miR-126 levels below the median exhibited a poorer survival rate, along with an elevated incidence of cardiovascular and renal events (Fourdinier et al., 2021). Recent findings by Scullion et al. (2021) have revealed that the serum levels of miR-126 were significantly lower in rats with nephrotoxic nephritis and individuals with acute endothelial and renal damage compared to healthy controls. There was a link between Sdc-1 and miR-126 in multivariate analysis. The endothelial glycocalyx (eGC) is a protective layer comprised of negatively charged polymers that coats the luminal surface of endothelial cells. It acts as a shield, safeguarding the vascular wall from potential damage that could trigger endothelial dysfunction. Sdc-1 shedding from eGC has been found to be elevated in individuals with chronic renal disease (Padberg et al., 2014). Interestingly, this was more prevalent among dialysis patients, indicating that this connection is connected to the degree of CKD (Liew et al., 2021). Sdc-1 mRNA upregulation predicts the development of acute damage in chronic renal disease (Jiang et al., 2021). Sdc-1 mRNA upregulation predicts the development of acute damage in chronic renal disease (Jiang et al., 2021). The research conducted by Cunha et al. (Fourdinier et al., 2021) solidified the notion of an association between miR-126 levels and endothelial dysfunction in a substantial cohort of patients with chronic kidney disease (CKD). In addition, it has been discovered that miR-126 promotes CXCL12-induced angiogenesis through its target gene Spred-1, indicating that the expression of miR-126 plays a role in angiogenesis and tissue regeneration (Bassand et al., 2021). MiR-126 suppression inhibited CXCL12-induced angiogenesis and migration in HUVEC endothelial cells. A notable correlation has been revealed between the cellular levels of miR-126 and the expression of Sdc-1 in prostate cancer cells. It has been observed that a reduction in Sdc-1 expression leads to a corresponding decrease in miR-126 expression (Fujii et al., 2015). MiR-126 reduces cell proliferation and promotes senescence in this model. IS and other protein-bound toxins have been shown to be harmful in the course of chronic renal disease. MiR-126 levels linked with free IS levels in multivariate studies. Higher levels of free IS have been linked to a higher relative risk of mortality when compared to total levels, suggesting that free IS levels are linked to miR-126 but not total IS (Liabeuf et al., 2011). Cunha et al. discovered that free IS enhanced IDI when the interaction was predictive, suggesting the additional predictive effect of this possible novel biomarker on critical CKD outcomes above and beyond existing prognostic indicators (e.g., baseline eGFR). Total pCG levels were also shown to be related to miR-126 but not free pCG levels. There was no link observed between free or total pCS. Cresol conjugation by bacteria yields mostly pCS and, to a lesser extent, pCG (Fourdinier et al., 2021). In a recent analysis of this cohort, free pCS was most linked with cardiovascular prognosis in non-dialyzed patients with chronic renal disease (Glorieux et al., 2021). IS is more effective in vascular remodelling, but pCS reduces endothelial cell survival. Moreover, Carmona et al. reported that IS triggers endothelial cells to create microvesicles and that the number of miRNAs in these microvesicles may be changed (Carmona et al., 2017). According to the findings of the identical research group, it has been revealed that microvesicles generated by pCS-treated endothelial cells impede the process of endothelial cell healing and expedite the aging of adult endothelial cells (Guerrero et al., 2020).

The equilibrium between vasoconstriction and vasodilation in vascular tone is regulated by factors released from the endothelium (Furchgott and Zawadzki, 1980). The levels of miR-142-3p in the blood are not only closely associated with vasodilatory markers in patients with end-stage renal disease (ESRD) and uremic mice, but also have a potential pathogenic function. By inhibiting the decrease of miR-142-3p expression, the restoration of ACh-mediated vasodilation occurs (Kétszeri et al., 2019). These findings are congruent with those of Sharma et al. (2012), who found that miR-142 plays an important role in adaptive cardiac overstretching in response to hemodynamic stress. According to Kétszeri et al. (2019), the presence of blood miR-142-3p serves as not only a biomarker for impaired renal function and endothelial dysfunction, but also as a direct facilitator of decreased ACh-mediated vasorelaxation in uremic patients. This suggests that targeting this molecule could potentially mitigate arterial stiffness and further complications in individuals with uremia. Uremia is characterized by prominent vascular lesions that affect the function of both VSMC and endothelial cells. These lesions have been closely associated with notable rates of morbidity and mortality due to cardiovascular complications. According to the data, it is evident that miRs play a crucial role in the development of uremic mesothelial sclerosis; however, further evidence is emerging regarding their potential involvement in vascular function impairment.

Endothelial dysfunction in atherosclerosis increases an inflammatory response to cholesterol and fat buildup inside the artery wall. The suppression of protective endothelium genes, specifically KLF2 and KLF4, through targeted targeting has revealed the critical role of miR-92a-3p as a regulator of angiogenesis and endothelial dysfunction (Wiese et al., 2019). Inhibiting miR-92a-3p in mice lacking the low-density lipoprotein receptor (Ldlr−/−) decreases the atherosclerotic load (Loyer et al., 2014). MiR-92a-3p levels in CKD sufferers’ blood are higher, and adenine-induced rise of aortic miR-92a-3p levels in CKD rats has been described (Shang et al., 2017). Therefore, impeding the activity of endothelial miR-92a-3p shows great potential as a treatment approach for atherosclerosis in chronic kidney disease. Wiese et al. (2019) conducted a study in which they administered LNA miRNA inhibitors to aortic endothelial cells *in vivo* via HDL, resulting in the discovery of a promising approach for reducing atherosclerosis in a renal injury model (ApoE−/−;5/6Nx). Specifically, the team found that simultaneous inhibition of miR-92a-3p and miR-489-3p yielded positive results in decreasing atherosclerosis. When compared to ApoE/control animals with undamaged kidneys, atherogenic miRNA (miR-92a-3p) levels were considerably higher in the vascular endothelium of ApoE/control mice. A recent research investigation has unveiled a novel miR-92a-3p/FAM220A/STAT3 pathway within human coronary artery epithelial cells (HCAEC) that exhibits potential in mitigating atherosclerosis in a rat model of renal injury. Wiese et al. (2019) discovered new miRNA targets, Tgfb2 and Fam220a, which were validated using real-time PCR and gene reporter experiments. Furthermore, there is a possibility that the presence of miR-92a-3p may have an impact on the pro-atherosclerotic activity of STAT3 through indirect means. This can be achieved by exerting direct control over FAM220A, a negative regulator of STAT3. According to numerous studies, STAT3 serves as a pro-inflammatory transcription factor within vascular endothelial cells, leading to the manifestation of vascular endothelial dysfunction and atherosclerosis (Lim and Fu, 2012; Dutzmann et al., 2015). Thus, dual inhibition of these miRNAs may alleviate atherosclerosis by activating the anti-inflammatory pathway (TGF) while inhibiting the pro-inflammatory route (STAT3). As a result, our data suggest the feasibility of using a dual inhibition strategy of LNA-based miRNAs to treat atherosclerosis in the context of CKD. It is evident that the use of endothelial miRNAs has promising prospects as a means of treating atherosclerosis and could potentially offer a more effective therapeutic approach for individuals with chronic kidney disease (CKD), especially those with advanced CKD who have not responded well to lipid-lowering therapies (see Table 3).

**TABLE 3:** Comparison of miRNAs in endothelial dysfunction.

miRNAs	Population/Methods	Function	Key-findings
miR-126 Barreto et al. (2009); Padberg et al. (2014)	Patient serum/Sprague-Dawley rats	miR-126 is enriched in endothelial cells and is a regulator of vascular integrity and angiogenesis. The -3p and -5p forms of miR-126 have activity in endothelial cells – 3p is anti-inflammatory and 5p is pro-proliferative。	● miR-126 induces CXCL12-induced angiogenesis through its target gene Spred-1, suggesting that miR-126 expression may be involved in angiogenesis and tissue regeneration. In HUVEC endothelial cells, CXCL12-induced angiogenesis and migration were abolished after miR-126 was inhibited
miR-142-3p Carmona et al. (2017); Guerrero et al. (2020)	p300 transgenic (p300tg) and wild-type (wt) mice/Patient serum	miR-142-3p is not only a biomarker of impaired renal function and endothelial dysfunction, but also a direct mediator of impaired acetylcholine-mediated vasodilation in uremia	● Downregulation of miR-142 is required to enable cytokine-mediated survival signalling during cardiac growth in response to haemodynamic stress and is a critical element of adaptive hypertrophy
			● Pharmacological exploitation of naturally occurring miRs and especially miR-142-3p could be a potential avenue to counteract vascular dysfunction in uremia and might thus ultimately prevent deleterious cardiovascular endpoints in patients with ESRD.
miR-92a-3p Furchgott and Zawadzki (1980)	Apolipoprotein E−/− (Apoe−/−) B6.129P2-Apoetm1Unc/J mice	miR-92a-3p has been identified as an important regulator of angiogenesis and endothelial dysfunction by targeting and repressing protective endothelial genes, including kruppel-like factors 2 (KLF2) and 4 (KLF4)	● Elevated aortic endothelial miRNA expression is associated with kidney injury and hypercholesterolemia. Inhibition of miR-92a-3p and miR-489-3p by a dual LNA treatment strategy significantly attenuated atherosclerosis and altered endothelial gene expression. The analysis identified Tgfb2 and Fam220a as novel target genes for miR-489-3p and miR-92a-3p, respectively ● In addition, FAM220A was demonstrated to be a direct target of miR-92a-3p in HCAEC, and the regulatory role of FAM220A on STAT3 phosphorylation in endothelial cells was verified. Thus, these findings support the potential of a dual LNA-based miRNA inhibition strategy for the treatment of atherosclerosis in CKD patients

## Summary and outlook

The focus of our investigation is the involvement of microRNAs (miRNAs) in cardiovascular problems associated with chronic kidney disease (CKD). The goal of this study is to highlight the significant promise of multiple miRNAs as new biomarkers and therapeutic targets in the setting of cardiovascular disease (CVD) associated with CKD. Numerous studies have offered persuasive data supporting the use of microRNAs (miRNAs) as effective biological markers of early vascular calcification, allowing for timely intervention to halt the evolution of this disease (Panizo et al., 2016). Additionally, microRNAs show significant promise as feasible targets for the prevention of arterial calcification and its associated negative cardiovascular consequences. Through the use of antisense miRNA inhibitors, the future treatment option for the management and prevention of vascular calcification is the suppression of the miRNAs that activate this process. This approach has greater promise than using nanocarrier-based approaches or vascular-specific carriers to reduce vascular calcification (Metzinger-Le Meuth et al., 2017). Chronic kidney disease (CKD) risk factors, including fibroblast growth factor-23, a uremic toxin, angiotensin II, and transforming growth factor-β, have been reported to inhibit the expression of cardiac miRNA-30. Nonetheless, the addition of additional miRNA-30 has shown a significant decrease in cardiomyocyte enlargement caused by the aforementioned variables. The findings establish a fresh conceptual framework for the pathophysiology of CKD-induced LVH while also identifying a potential focal area for therapeutic intervention aimed at reducing LVH in CKD patients (Bao et al., 2021). MiRNAs have the potential to become sensitive and specific biomarkers for renal disorders due to their tissue specificity and stability in a variety of biological components. The constant development of new perspectives in the domain of microRNAs (miRNAs) is a long-term result of the continued use of animal models, *in vitro* tests, and human subject research. However, the real challenges are in translating these scientific insights into accurate clinical diagnostic tools. Numerous intriguing non-invasive miRNA biomarkers have been discovered in the realm of kidney disease, holding promise for improving prognosis prediction and diagnostic precision, while also monitoring disease progression and treatment efficacy. Individuals with differing degrees of chronic kidney disease (CKD) and those receiving hemodialysis therapy have different levels of expression of the cellular regulatory molecules known as miRNAs. Patients with chronic kidney disease (CKD) stages III-V who are receiving hemodialysis had elevated expressions of miR-143, miR-145, and miR-223. In contrast, lower amounts of these microRNAs have been seen in kidney transplant patients. In contrast, miR-126 and miR-155 can be identified by their increased abundance in people with CKD stages III-V, followed by a decrease in manifestation among those on hemodialysis, and finally by a more pronounced decrease in renal transplant recipients. These observed differences in miRNA levels illustrate the complex interaction between physiological perturbations and the resulting alteration of gene regulatory systems. This highlights miRNAs’ potential as viable diagnostic and therapeutic targets in the management of CKD and its subsequent treatment approaches (Franczyk et al., 2022). Significant clinical trials with a broad patient population, on the other hand, are necessary to test miRNAs’ clinical relevance and confirm their accuracy and prevalence. In conclusion, there is a hypothesis indicating the involvement of miRNA in clinical diagnosis; nevertheless, additional research is required. As we move forward, it is extremely likely that microRNAs (miRNAs) will provide significant advantages to current advances in diagnostics and therapies, notably in chronic kidney disease (CKD) and its related cardiovascular illnesses (CVD). MiRNAs are poised to serve a critical and vital function in this respect.
